# Postmarket safety profile of suicide/self-injury for GLP-1 receptor agonist: a real-world pharmacovigilance analysis

**DOI:** 10.1192/j.eurpsy.2023.2474

**Published:** 2023-11-30

**Authors:** Congqin Chen, Rijing Zhou, Fang Fu, Jie Xiao

**Affiliations:** 1 Department of Pharmacy, Xiamen Cardiovascular Hospital, Xiamen University, Xiamen, China; 2 Department of Intensive Care Unit, Xiamen Cardiovascular Hospital, Xiamen University, Xiamen, China

**Keywords:** FAERS, GLP-1RA, pharmacovigilance analysis, self-injury, suicide

## Abstract

**Background:**

Recent reports of individuals experiencing suicidal and/or self-injurious behaviors while using liraglutide and semaglutide have heightened the concerns regarding neuropsychiatric safety of Glucagon-like peptide-1 agonists (GLP-1RAs). As real-world evidence is very limited, we explored the association between GLP-1RA and suicide/self-injury by mining the FDA Adverse Event Reporting System (FAERS) database.

**Methods:**

The FAERS database was queried from 2005 Q2 to 2023 Q2. The Reporting Odds Ratio (ROR) and Empirical Bayes Geometric Mean (EBGM) were used to conduct the disproportionality analysis.

**Results:**

A total of 534 GLP-1RA-associated suicide/self-injury cases were reported in the FAERS during the study period. GLP-1RA did not cause a disproportionate increase in overall suicidal and self-injurious cases (ROR: 0.16, 95%CI 0.15-0.18, P < 0.001; EBGM05: 0.15). Stratified analyses found no safety signal of suicide/injury for GLP-1RA in both females and males. The ROR for suicide/self-injury with GLP-1RA was slightly elevated (ROR: 2.50, 95%CI 1.02-6.13, P = 0.05) in children, while the EBGM05 was < 2 in this population. No significant signal value was observed in other age groups. No over-reporting of suicide/self-injury was identified for GLP-1RA before or after the COVID-19 pandemic outbreak.

**Conclusions:**

The cases of suicide or self-injury reported to FAERS do not indicate any overall safety signal attributable to GLP-1RA at this time. Subgroup analysis revealed a marginal elevation of ROR for suicide and self-injury with GLP-1RA in children, but no safety signal was detected by EBGM05 in this population. Further large-scale prospective investigations are still warranted to further confirm this finding.

## Introduction

Glucagon-like peptide-1 receptor agonists (GLP-1RAs) are a class of medications that mimic the action of the natural hormone GLP-1, which plays a crucial role in regulating glucose metabolism and various other physiological processes [1, 2]. GLP-1RA has gained a sudden surge in popularity for its ability to trigger glycemic control, weight loss, and cardiovascular risk reduction [3–6]. Recently, rare but serious psychiatric adverse events (AEs) associated with GLP1-RA, such as suicide and self-injury, have gained attention [7]. The Icelandic Medicines Agency’s alert regarding reports of suicidal thoughts and self-injury in individuals using liraglutide and semaglutide has raised concerns about the potential suicidal and/or self-injurious risk associated with GLP-1RA [8]. Although causality cannot be proved based on the case anecdotes, constant vigilance for this phenomenon is warranted.

Despite significant concerns raised by recent case reports, the evidence of GLP-1RA-associated suicidal and self-injurious risk is very limited. Wilding and colleagues pooled data from weight-management clinical trials to assess the neuropsychiatric safety of liraglutide [7]. The results of this exploratory pooled analysis revealed a slight numerical imbalance in suicidal ideation with liraglutide through AE reporting, while no imbalance was noted through prospective questionnaire assessments. The analysis is restricted to obese patients, and the drug under consideration is specifically liraglutide 3.0 mg. While safety assessments in clinical trials offer insights into AEs within the indicated population, these studies have limitations due to their duration and predefined patient criteria. Consequently, potential safety concerns may emerge in real-world settings that were not apparent during clinical trial evaluations [9]. No substantial evidence has been accumulated to address the uncertainty regarding the association of GLP-1RA with suicide or self-injury to date. Therefore, in order to fully understand the potential risk of suicide/self-injury related to GLP-1RA, a postmarket pharmacovigilance study was carried out to explore the association between GLP-1RA and suicidal/self-injury reports by utilizing the Food and Drug Administration (FDA) Adverse Event Reporting System (FAERS) database.

## Methods

### Data source

Managed by the FDA, FAERS compiles AE reports from various contributors such as healthcare professionals, patients, drug manufacturers, and more [10–12]. AE symptoms are categorized using the globally accepted and clinically validated Medical Dictionary for Regulatory Activities (MedDRA) terminology [13]. FAERS enables the examination of unforeseen AE trends that might go unnoticed in clinical trials due to participant limitations [14, 15].

### Data queries

**Figure 1.**
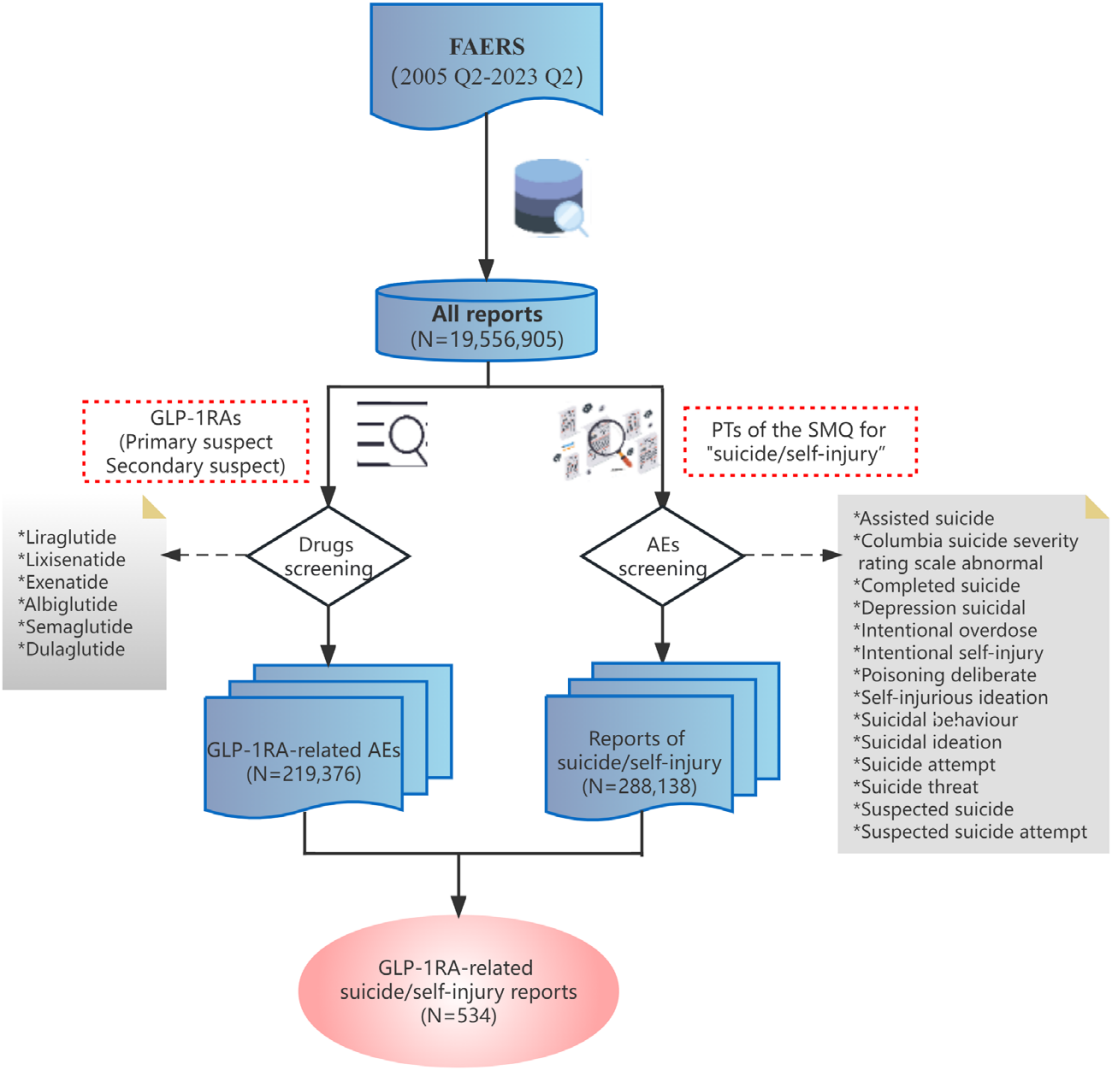
Flow chart of data queries within the FAERS database. FAERS, the U.S. Food and Drug Administration (FDA) Adverse Event Reporting System; Q2, the second quarter; GLP-1RA, glucagon-like peptide-1 receptor agonist; PT, preferred term; SMQ, standard MedDRA (Medical Dictionary for Regulatory Activities) query.

In accordance with the aforementioned, the recent reports of individuals exhibiting suicidal and self-injurious behaviors while using liraglutide and semaglutide have sparked concerns. Therefore, the FAERS database was queried from the second quarter (Q2) of 2005 to the Q2 of 2023, to analyze disproportionality and investigate the association between GLP-1RA and AEs involving suicide and self-injury. Each report was classified based on the contingency table (Table 1), where “*a*” represents the number of suicide/self-injury reports for GLP-1RA, “*b*” represents the reports for GLP-1RA without suicide/self-injury, “*c*” represents the number of suicide/self-injury reports for all other drugs, and “*d*” represents the reports for all other drugs without suicide/self-injury. Reports associated with GLP-1RA were identified when any of the six GLP-1RAs (liraglutide, lixisenatide, exenatide, albiglutide, semaglutide, and dulaglutide) was classified as “primary suspected” or “secondary suspected” drug in reported cases. Suicidal and self-injurious reports were queried by combining the MedDRA 26.0 preferred terms (PTs) of the standard MedDRA query (SMQ) for “suicide/self-injury.” The precise terms used were “assisted suicide,” “Columbia suicide severity rating scale abnormal,” “completed suicide,” “depression suicidal,” “intentional overdose,” “intentional self-injury,” “poisoning deliberate,” “self-injurious ideation,” “suicidal behavior,” “suicidal ideation,” “suicide attempt,” “suicide threat,” “suspected suicide,” “suspected suicide attempt.” Utilization of globally accepted and clinically validated mesh terms enabled us to effectively address the reports pertaining to the objectives of this study. The detailed data processing procedure is shown in Figure 1.

**Table 1.** Contingency table for disproportionality analysis

	Event suicide/self-injury	All other events
GLP-1RA	*a*	*b*
All other drugs	*c*	*d*

GLP-1RA, glucagon-like peptide-1 receptor agonist.

### Data analysis

A population-based pharmacovigilance study utilizing a case/non-case approach was employed to investigate the potential association between GLP-1RA and reports of suicidal tendencies and/or self-injury. This method is a prevalent technique employed in pharmacovigilance research to detect safety signals [16–18]. From a mathematical perspective, the concept behind the case/non-case approach involves contrasting the reporting rate of a specific AE in patients who have been exposed to a particular drug with the reporting rate of the same AE in patients who haven’t been exposed to that drug [19, 20]. In this study, disproportionality was evaluated by the empirical Bayes geometric mean (EBGM) from multi-item gamma Poisson shrinker (MGPS) and the reporting odds ratio (ROR). Signal was defined when the EBGM05 metric, a lower one-sided 95% confidence limit of the EBGM ≥ 2.0 [20] or the lower limits of the 95% confidence intervals (95% CIs) of ROR exceeded 1 in at least three reports [21]. The equations of EBGM and ROR are listed in Table 2.

**Table 2.** Equations and criteria for ROR and MGPS

Algorithms	Equation	Criteria
ROR	ROR = *ad*/*b*/*c*	Lower limit of 95% CI > 1, *N* ≥ 3
	95% CI = e^ln(ROR) ± 1.96(1/*a* + 1/*b* + 1/*c* + 1/*d*)^0.5^	
MGPS	EBGM = *a*(*a* + *b* + *c* + *d*)/(*a* + *c*)/(*a* + *b*)	EBGM05 > 2
	95% CI = e^ln(EBGM) ± 1.96(1/*a* + 1/*b* + 1/*c* + 1/*d*)^0.5^	

*a*, the number of suicide/self-injury reports for GLP-1RA; *b*, the reports for GLP-1RA without suicide/self-injury; *c*, the number of suicide/self-injury reports for all other drugs; *d*, the reports for all other drugs without suicide/self-injury; 95% CI, 95% confidence interval; *N*, the number of reports; ROR, the reporting odds ratio; MGPS, multi-item gamma Poisson shrinker; EBGM, empirical Bayesian geometric mean; EBGM05, the lower limit of 95% CI of EBGM; GLP-1RA, glucagon-like peptide-1 receptor agonist.

## Results

### Data overview of adverse events reported in the FAERS database, 2005Q2–2023Q2

Over the study period, a total of 19,556,905 de-duplicated records were extracted from the FAERS database. Overall, 219,376 AEs were found to be related to GLP-1RA, and 288,138 reports related to suicide/self-injury were documented. Among them, GLP-1RA was identified as the suspected drug causing suicide/self-injury in 534 reports.

### Descriptive analysis of suicidal and self-injurious adverse events among GLP-1RA users

The clinical characteristics of GLP-1RA-associated suicide/self-injury are presented in Table 3. Within this cohort, 53.18% were female and 41.20% were male. Cases of GLP-1RA-associated suicide/self-injury were more likely to be reported in adults aged 25 to 65 years (48.50%). Customers reported 38.58% of the cases, physicians reported 34.46% of the cases, pharmacists reported 9.55% of the cases, and 8.61% of the cases were reported by other healthcare providers. Most of the suicide/self-injury AEs related to GLP-1RA were reported during the first quarter of 2020 to the second quarter of 2023. After analyzing all the reported suicide/self-harm cases related to GLP-1RA, it was found that 51.50% exhibited symptoms of suicidal ideation, 19.29% had attempted suicide, and 18.73% experienced intentional overdose.

**Table 3.** Clinical characteristics of suicide/self-injury cases reported to FAERS during the study period

Clinical characteristics	GLP-1RA-associated suicide/self-injury reports (*N* = 534)	Other drugs-associated suicide/self-injury reports (*N* = 287,604)	All suicide/self-injury reports (*N* = 288,138)
*Gender*			
Male	220 (41.20%)	109,118 (37.94%)	109,338 (37.95%)
Female	284 (53.18%)	153,589 (53.40%)	153,873 (53.40%)
Unspecified	30 (5.62%)	24,897 (8.66%)	24,927 (8.65%)
*Age (years)*			
Children (0–14)	5 (0.94%)	9,336 (3.25%)	9,341 (3.24%)
Young adults (15–24)	25 (4.68%)	35,322 (12.28%)	35,347 (12.27%)
Adults (25–65)	259 (48.50%)	145,694 (50.66%)	145,953 (50.65%)
Elderly (≥66)	61 (11.42%)	25,293 (8.79%)	25,354 (8.80%)
Unspecified	184 (34.46%)	71,959 (25.02%)	72,143 (25.04%)
*Reporting sources*			
Physician	184 (34.46%)	94,478 (32.85%)	94,662 (32.85%)
Pharmacist	51 (9.55%)	20,534 (7.14%)	20,585 (7.14%)
Other healthcare providers	46 (8.61%)	54,571 (18.97%)	54,617 (18.96%)
Customers	206 (38.58%)	71,596 (24.89%)	71,802 (24.92%)
Lawyer	1 (0.19%)	13,322 (4.63%)	13,323 (4.62%)
Sales	0 (0.00%)	0 (0.00%)	0 (0.00%)
Unspecified	46 (8.61%)	33,103 (11.51%)	33,149 (11.50%)
*Reporting year*			
2005Q2–2007Q4	25 (4.68%)	24,685 (8.58%)	24,710 (8.58%)
2008Q1–2011Q4	70 (13.11%)	56,584 (19.67%)	56,654 (19.66%)
2012Q1–2015Q4	70 (13.11%)	54,842 (19.07%)	54,912 (19.06%)
2016Q1–2019Q4	129 (24.16%)	80,332 (27.93%)	80,461 (27.92%)
2020Q1–2023Q2	240 (44.94%)	71,161 (24.74%)	71,401 (24.78%)
*PTs*			
Assisted suicide	0 (0.00%)	41 (0.01%)	41 (0.01%)
Columbia suicide severity rating scale abnormal	0 (0.00%)	2 (<0.01%)	2 (<0.01%)
Completed suicide	50 (9.36%)	77,900 (27.09%)	77,950 (27.05%)
Depression suicidal	24 (4.49%)	3,453 (1.20%)	3,477 (1.21%)
Intentional overdose	100 (18.73%)	59,015 (20.52%)	59,115 (20.52%)
Intentional self-injury	12 (2.25%)	23,450 (8.15%)	23,462 (8.14%)
Poisoning deliberate	12 (2.25%)	6,227 (2.17%)	6,239 (2.17%)
Self-injurious ideation	7 (1.31%)	4,163 (1.45%)	4,170 (1.45%)
Suicidal behavior	8 (1.50%)	4,567 (1.59%)	4,575 (1.59%)
Suicidal ideation	275 (51.50%)	94,409 (32.83%)	94,684 (32.86%)
Suicide attempt	103 (19.29%)	61,320 (21.32%)	61,423 (21.32%)
Suicide threat	0 (0.00%)	293 (0.10%)	293 (0.10%)
Suspected suicide	5 (0.94%)	2,498 (0.87%)	2,503 (0.97%)
Suspected suicide attempt	0 (0.00%)	170 (0.06%)	170 (0.06%)

GLP-1RA, glucagon-like peptide-1 receptor agonist; PT, preferred term.

### Disproportionality analysis for the association of suicide/self-injury with GLP-1RA

**Figure 2.**
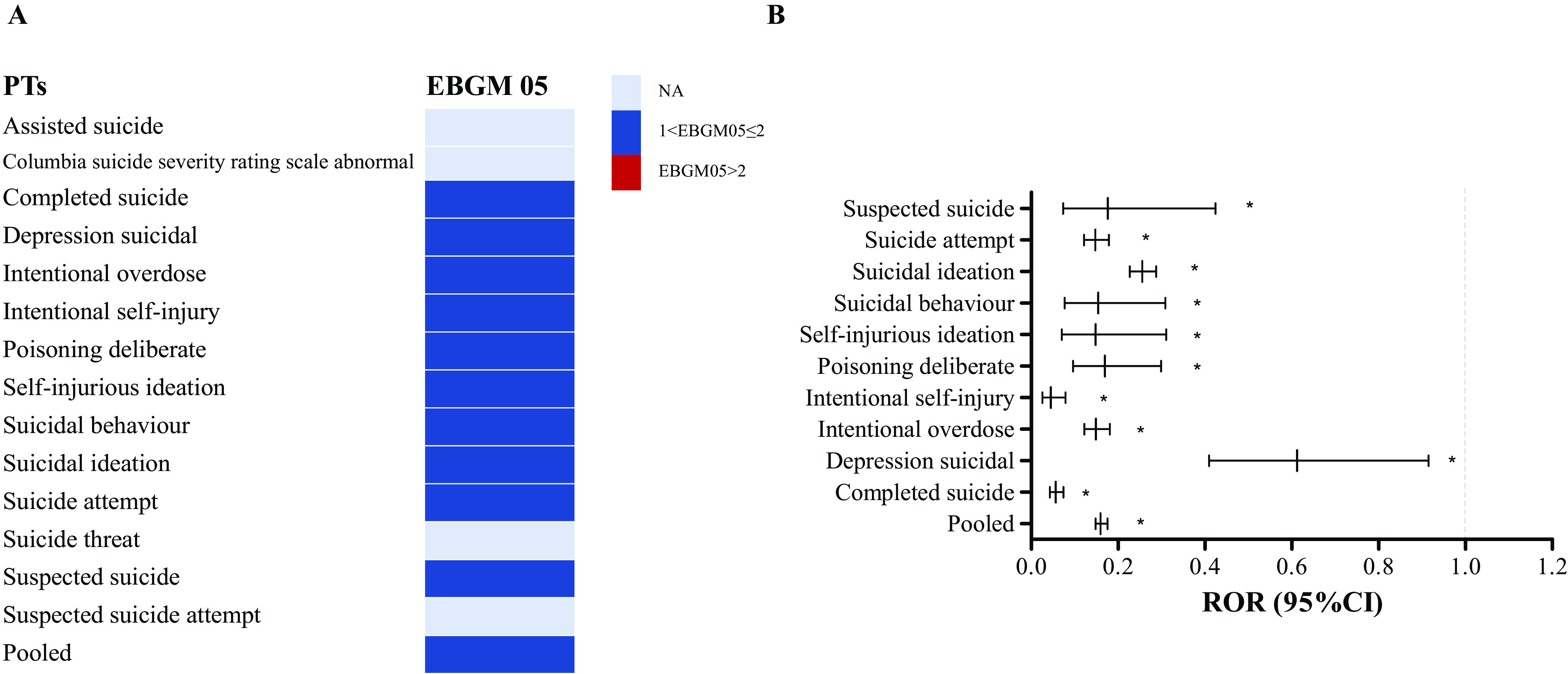
Results of overall disproportionality analysis for GLP-1RA-associated suicide/self-injury at PT levels. (A) EBGM05s of GLP-1RA-associated suicide/self-injury. (B) RORs (95% CI) of GLP-1RA-associated suicide/self-injury. EBGM05, lower one-sided 95% confidence limit (95% CI) of the empirical Bayes geometric mean; PT, preferred term; ROR, reporting odds ratio; GLP-1RA, glucagon-like peptide-1 receptor agonist.

The results of overall disproportionality analysis are summarized in Figure 2. The analysis demonstrated that no over-reporting of suicide/self-injury was identified in GLP-1RA, with a pooled ROR of 0.16 (95% CI 0.15–0.18, *P* < 0.001) and EBGM05 of 0.15. We calculated the disproportional pattern of each suicidal and self-injurious PT with GLP-RA. No safety signal was detected for all related PTs.

**Figure 3.**
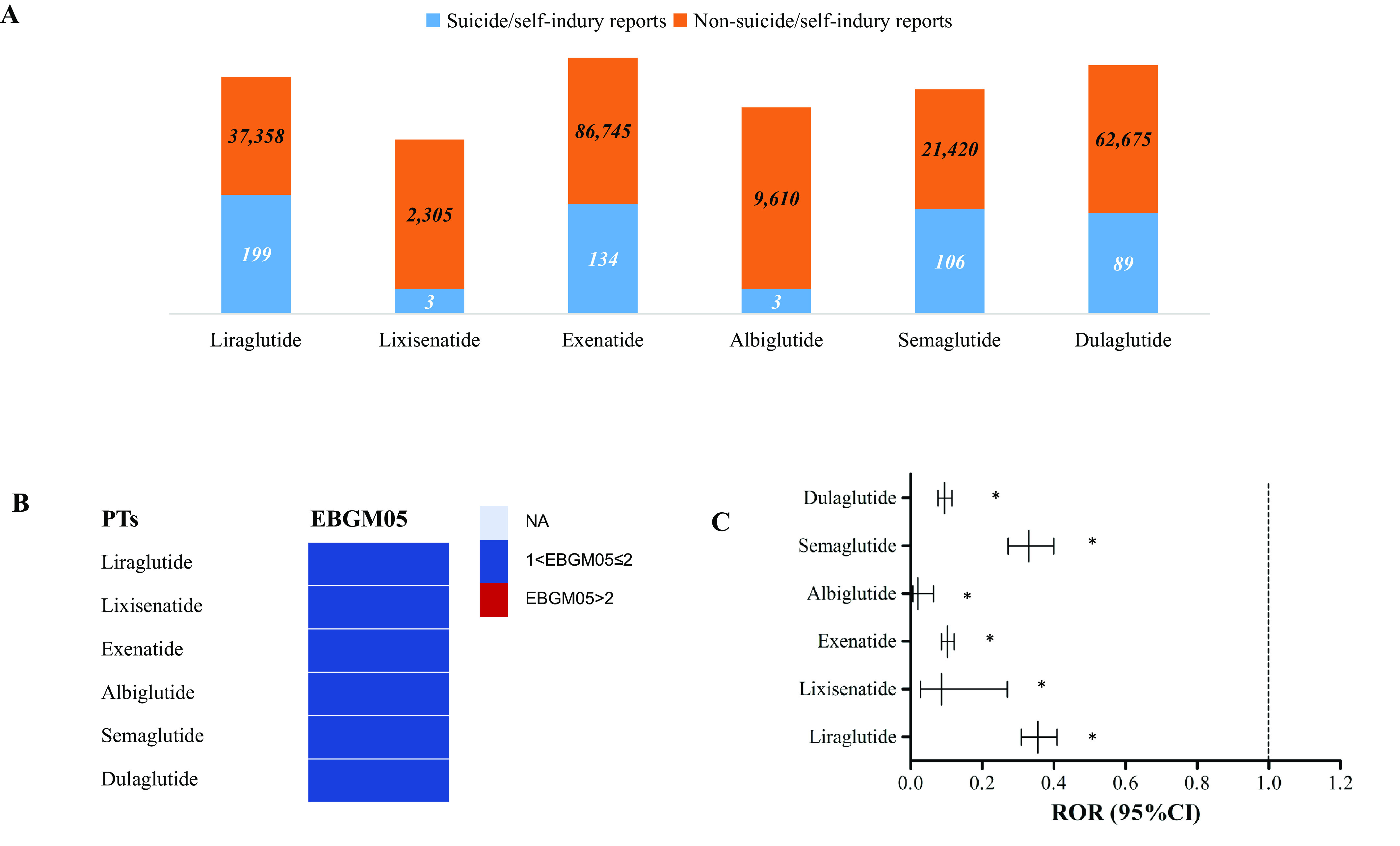
Results of disproportionality analysis for suicidal and self-injurious reports associated with GLP-1RA at drug levels. (A) Number of suicide/self-injury and non-suicide/self-injury reports for each GLP-1RA drug. (B) EBGM05 of GLP-1RA-associated suicide/self-injury for each distinct GLP-1RA drug. (C) RORs (95% CI) of GLP-1RA-associated suicide/self-injury for each distinct GLP-1RA drug. ROR, reporting odds ratio; GLP-1RA, glucagon-like peptide-1 receptor agonist; EBGM05, lower one-sided 95% confidence limit (95% CI) of the empirical Bayes geometric mean.

Figure 3A displays the number of reports for each specific GLP-1RA drug. It can be observed that among the suicidal and self-injurious reports related to GLP-1RA, 199 cases were associated with liraglutide, 134 cases with exenatide, 106 cases with semaglutide, 89 cases with dulaglutide, 3 cases with albiglutide, and 3 cases with lixisenatide. Stratified analyses revealed low ROR and EBGM05 value for suicide/self-injury in all GLP-1RAs, implying that none of the six GLP-1RA drugs examined in this study were linked to the risk of suicide and/or self-injury in general (Figure 3B,C).

**Figure 4.**
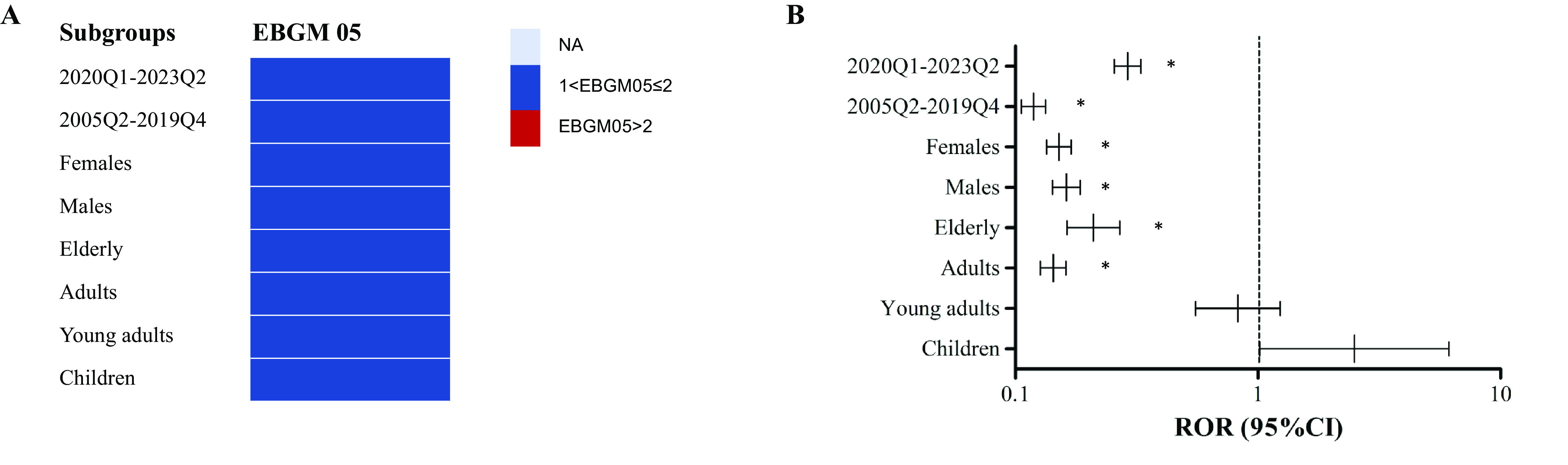
Results of subgroup disproportionality analysis of GLP-1RA-associated suicide/self-injury based on age, sex and reporting time. (A) EBGM05s of GLP-1RA-associated suicide/self-injury in different sex, age and reporting time groups. (B) RORs (95% CI) of GLP-1RA-associated suicide/self-injury in different sex, age and reporting time groups. ROR, reporting odds ratio; 95% CI, 95% confidence limit; GLP-1RA, glucagon-like peptide-1 receptor agonist; EBGM05, lower one-sided 95% CI of the empirical Bayes geometric mean.

To delve deeper into individual characteristics, separate sub-analyses were conducted based on gender, age, and reporting time (Figure 4). No safety signal of suicide/injury for GLP-1RA was detected in both females (ROR 0.15, 95% CI 0.13–0.17, *P* < 0.001; EBGM05 0.14) and males (ROR 0.16, 95% CI 0.14–0.19, *P* < 0.001; EBGM05 0.15). Stratified analysis by age showed that the ROR for suicide/self-injury with GLP-1RA was slightly elevated in children (ROR 2.50, 95% CI 1.02–6.13, *P* = 0.05), while the EBGM05 of GLP-1RA-associated suicide/self-injury was lower than 2 for children. No significant signal value was observed in other age groups based on the results of ROR and EBGM05. Using the fourth quarter of 2019 and the first quarter of 2020 as the cut-offs, we divided the reports into two different groups based on the time of COVID-19 pandemic outbreak. Among the reports of suicide/self-injury with GLP-1RA, 294 (55.06%) cases were reported in 2005Q2–2019Q4, and 240 (44.94%) cases were reported during 2020Q1–2023Q2. No over-reporting of suicide/self-injury was identified for GLP-1RA in the two periods.

## Discussion

The neuropsychiatric safety of GLP-1RAs has garnered attention, primarily owing to their centrally acting appetite-suppressing activity [7]. Concerns have escalated following recent reports of individuals exhibiting suicidal and self-injurious behaviors while using liraglutide and semaglutide [8]. The available evidence on the association between GLP-1RA and the risk of suicidal and self-injurious behaviors is quite limited. Wilding and his colleagues [7] conducted a comprehensive analysis by combining data from various weight-management clinical trials [22–27], focusing on the neuropsychiatric safety of liraglutide. The outcomes of this exploratory pooled analysis provide a level of reassurance regarding the suicidal safety associated with the use of liraglutide 3.0 mg in patients who share similarities with those involved in the examined trials. Safety assessments conducted in clinical trials provide valuable insights into AEs within a specified population. However, these studies have inherent limitations, primarily stemming from their controlled duration and predefined patient criteria [22–26]. The controlled nature of clinical trials may not fully capture the diverse and dynamic conditions encountered in real-world settings. As a result, potential safety concerns might arise in everyday clinical practice that were not evident during the more controlled and structured environment of clinical trial evaluations [28]. Therefore, postmarketing surveillance and real-world evidence play a crucial role in continuously monitoring the safety and effectiveness of medications once they are in widespread use [29–31]. The analysis of pharmacovigilance for a drug, utilizing postmarketing safety databases like the FAERS, plays a crucial role in the ongoing management of risks by detecting safety signals that may not have been identified during the drug’s clinical development phase [10, 14, 32–35]. A previous pharmacovigilance study based on FAERS conducted an analysis of the potential risk between GLP-1RA and tumors and provided new real-world evidence for oncology safety information of GLP-1RA [12]. To the best of our knowledge, our study is the first pharmacovigilance study of suicide/self-injury AEs associated with GLP-1RAs based on the FAERS database. The results of our analysis did not reveal any abnormally elevated association of GLP-1RA with overall cases of suicide/self-injury, whether based on the SMQ level or focusing on specific subcategories at the PT level. This suggests that, based on the available data, there is no evidence to suggest a heightened risk of suicide or self-injury associated with the use of GLP-1RA. These findings contribute to the understanding of the safety profile of GLP-1RA in relation to these specific AEs.

We identified a total of 534 cases of suicidal and self-injurious behavior associated with GLP-1RAs in the FAERS database during the period from 2005Q2 to 2023Q2. Among these cases, 53.18% were female and 41.20% were male. Epidemiological data on suicide reveals that the 12-month prevalence of suicidal ideation is greater in females than males whereas the prevalence of suicide attempts is comparable for females and males [36]. Among the 534 cases of suicidal and self-injurious behavior associated with GLP-1RA in our study, 275 (51.50%) cases experienced suicidal ideation. This could potentially contribute to the higher proportion of females in all these cases. Following a subgroup disproportionality analysis based on gender, the findings indicate that no safety signals were identified for either females or males. This suggests that, within the studied population, there were no notable differences in the occurrence of suicidal and self-injurious AEs between the two genders that would raise concerns requiring further investigation.

In response to the Icelandic Medicines Agency’s alert regarding reports of suicidal thoughts and self-injury linked to the use of liraglutide and semaglutide, we undertook a comprehensive analysis to assess the risk of suicide/self-injury associated with various GLP-1RAs. Through a subgroup analysis based on the specific drug, the results revealed that none of the six GLP-1RA drugs examined generated a safety signal in relation to suicide or self-injury. This outcome suggests that, despite the specific concerns raised in the alert for liraglutide and semaglutide, the broader class of GLP-1RAs does not exhibit an elevated risk of these AEs based on the available data. The subgroup analysis enhances our understanding of the safety profile of individual GLP-1RA drugs in the context of suicide and self-injury.

Suicidal and self-injurious patterns linked to age differ among global regions. According to the results of a national survey conducted in 2015, the annual rate of suicide attempts is three to five times greater in young adults aged 18–25 years, compared with older age groups in the United States [37]. The Office for National Statistics in UK detected an increase in suicide rates in children and young people in 2014 [38]. We conducted a subgroup analysis based on age to assess the proportional imbalance of AEs related to suicide/self-injury for GLP-1RA within different age groups. Specifically, in the subgroup of children, the ROR indicated a slight elevation in the reporting pattern of suicide/self-injury. However, it is essential to interpret this result with caution, as the EBGM05 for GLP-1RA-associated suicide/self-injury in children was lower than 2, suggesting a lack of robust signal strength. Contrastingly, no statistically significant signal values were observed in other age groups, including younger adults, adults, and the elderly, based on both ROR and EBGM05. Compared to adults, relatively few studies have investigated the use of GLP-1RA in pediatric patients with diabetes and obesity. However, some studies have provided strong evidence for the beneficial effects of GLP-1RA in children with type 2 diabetes mellitus (T2D) and obesity, and have paved the way for GLP-1RA to be introduced to pediatric clinical practice [39–42]. Some GLP-1RAs have been approved in pediatric population several years ago. In June 2019, The FDA approved liraglutide for the treatment of pediatric patients over the age of 10 years with T2D. In December 2020, the FDA also approved liraglutide for treatment of obesity in adolescents 12–17 years of age. In July 2021, an extended-release formulation of exenatide was approved by FDA in patients ≥10 years old. In November 2022, dulaglutide was indicated as an adjunct to diet and exercise to improve glycemic control in adults and pediatric patients ≥10 years of age with type 2 diabetes mellitus. In December 2022, semaglutide was also indicated for chronic weight management in pediatric patients aged 12 years and older. While there is a marginal elevation of ROR in children, the discrepancy with the EBGM05 underscores the complexity of signal detection. Even our analytical period contains 4 years of GLP-1RA usage in pediatric population, it is still crucial for future research to explore the underlying mechanisms contributing to this observed association and to validate findings with larger and more diverse datasets.

The COVID-19 pandemic has had a significant impact on the mental well-being of people worldwide. Various factors associated with the COVID-19 pandemic, such as social isolation, economic stress, uncertainty, and disruptions to daily life, were identified as potential contributors to mental health challenges. These challenges, in turn, could be linked to an increased risk of suicidal thoughts and behaviors [43, 44]. We divided the reports of suicide and self-injury associated with GLP-1RA into two groups based on the time of the COVID-19 pandemic outbreak. Among the cases reported, 55.06% occurred from the second quarter of 2005 to the fourth quarter of 2019, while 44.94% cases were reported during the first quarter of 2020 to the second quarter of 2023. Notably, there was no identified over-reporting of suicide or self-injury for GLP-1RA in either of the two periods. During the pandemic, more adverse reactions related to suicide in individuals using GLP-1RA reported may be attributed to various factors. Notably, in June 2021, the FDA approved semaglutide for chronic weight management in adults dealing with general obesity or overweight and presenting at least one weight-related condition [45]. The introduction of this new indication may have led to a broader user population, consequently resulting in a higher number of reported adverse reactions.

With the increasing popularity of GLP-1RA, especially following its approval for obesity indications, a growing number of individuals may opt for GLP-1RA, potentially involving off-label use. We investigated pre-July 2017 FAERS data, predating the approval of GLP-1RA for pediatric use, and identified 80 cases of AEs in children related to GLP-1RA, suggesting the existence of off-label use. It is crucial to emphasize that off-label use must be justified by specific clinical needs, supported by scientific evidence from evidence-based medicine, and administered under close medical supervision [46, 47]. Individuals should refrain from unauthorized off-label use, as it poses significant risks [48]. Each GLP-1RA has explicit indications and contraindications for weight loss [49]. Blindly following trends in usage without adhering to specific requirements and medical supervision is strongly discouraged.

The utilization of the FAERS in pharmacovigilance is indispensable, yet its application is constrained by several notable limitations. Firstly, the majority of reports lack evidence establishing a causal link between the reported AE and drug exposure. The inability to definitively establish causality is a constraint shared by all pharmacovigilance studies and observational cohort studies [50, 51]. Secondly, cases in FAERS might contain incomplete information such as dose, complication, onset time, and other details. The incomplete capture of AEs hampers the comprehensiveness of the database, rendering it challenging to obtain a holistic understanding of a drug’s safety profile. Given the limitations mentioned, our study cannot provide a comprehensive assessment of the correlation between GLP-1RA dosage and indication with suicide/self-injury, and it also lacks the capability to thoroughly evaluate other potential risk factors or comorbidities. Furthermore, reporting bias is a significant concern, potentially skewing the perception of drug safety by overemphasizing widely publicized or severe events. Therefore, any conclusions drawn from pharmacovigilance analysis should be interpreted within the context of these constraints, and further research with a broader scope may be necessary to elucidate a more complete understanding of the relationships involved. Notwithstanding these limitations, disproportionality analysis still remains a crucial tool for detecting potential safety signals associated with drugs and guiding further investigations [16, 29, 33, 52]. It is important to acknowledge that our study represents a preliminary snapshot based on available data and methodologies. Future research endeavors could consider employing additional approaches, such as prospective clinical trials or evidence-based cohort studies, to overcome some of the limitations associated with FAERS data.

## Conclusions

Concerns regarding the potential risk of suicide or self-injury associated with GLP-1RA have been raised based on anecdotal case reports. This study contributes postmarket evidence on the neuropsychiatric safety profile of GLP-1RA. Analysis of suicide and self-injury cases reported to FAERS currently does not indicate any safety concerns directly attributable to GLP-1RA. Subgroup analysis has revealed a marginal increase in the ROR for suicide and self-injury associated with GLP-1RA use in children, but no safety signal was detected using EBGM05 in this specific population. Nevertheless, it is essential to emphasize that our study represents a preliminary snapshot based on available data and methodologies. To substantiate and enhance these initial findings, there is a pressing need for more comprehensive prospective investigations conducted on a larger scale.
